# Different clinical, virological, serological and tissue tropism outcomes of two new and one old Belgian type 1 subtype 1 porcine reproductive and respiratory virus (PRRSV) isolates

**DOI:** 10.1186/s13567-015-0166-3

**Published:** 2015-03-21

**Authors:** Ilias S Frydas, Ivan Trus, Lise K Kvisgaard, Caroline Bonckaert, Vishwanatha RAP Reddy, Yewei Li, Lars E Larsen, Hans J Nauwynck

**Affiliations:** Laboratory of Virology, Department of Virology, Immunology and Parasitology, Faculty of Veterinary Medicine, Ghent University, Salisburylaan 133, B-9820 Merelbeke, Ghent, Belgium; National Veterinary Institute, Technical University of Denmark, Frederiksberg C, Denmark

## Abstract

In this study, the pathogenic behavior of PRRSV 13V091 and 13V117, isolated in 2013 from two different Belgian farms with enzootic respiratory problems shortly after weaning in the nursery, were compared with the Belgian strain 07V063 isolated in 2007. Full-length genome sequencing was performed to identify their origin. Twelve weeks-old pigs were inoculated intranasally (IN) with 13V091, 13V117 or 07V063 (9 pigs/group). At 10 days post inoculation (dpi), 4 animals from each group were euthanized and tissues were collected for pathology, virological and serological analysis. 13V091 infection resulted in the highest respiratory disease scores and longest period of fever. Gross lung lesions were more pronounced for 13V091 (13%), than for 13V117 (7%) and 07V063 (11%). The nasal shedding and viremia was also most extensive with 13V091. The 13V091 group showed the highest virus replication in conchae, tonsils and retropharyngeal lymph nodes. 13V117 infection resulted in the lowest virus replication in lymphoid tissues. 13V091 showed higher numbers of sialoadhesin^−^ infected cells/mm^2^ in conchae, tonsils and spleen than 13V117 and 07V063. Neutralizing antibody response with 07V063 was stronger than with 13V091 and 13V117. It can be concluded that (i) 13V091 is a highly pathogenic type 1 subtype 1 PRRSV strain that replicates better than 07V063 and 13V117 and has a strong tropism for sialoadhesin^−^ cells and (ii) despite the close genetic relationship between 13V117 and 07V063, 13V117 has an increased nasal replication and shedding, but a decreased replication in lymphoid tissues compared to 07V063.

**Electronic supplementary material:**

The online version of this article (doi:10.1186/s13567-015-0166-3) contains supplementary material, which is available to authorized users.

## Introduction

Porcine reproductive and respiratory syndrome (PRRS) is a devastating disease that continuously hits swine industry all over the world [1]. A recent economic analysis in the US demonstrated that the amount of total annual losses increased from $560 million in 2005 to $664 million in 2011 [2]. The disease first appeared in the US in the late eighties and then a few years later in Europe and Asia [3]. The causative agent is a positive single-stranded enveloped RNA virus classified together with lactate dehydrogenase virus (LDV), simian hemorrhagic fever virus (SDHF) and equine arteritis virus (EAV) in the family of the *Arteriviridae* within the order of the *Nidovirales* [4]. PRRSV causes reproductive failure in late-term gestation sows with an increased number of stillborn, mummified and weak-born piglets. Respiratory disorders are characterized mainly by dyspnea and tachypnea in young animals [5,6].

The PRRSV genome is approximately 15 kb in length and contains nine open reading frames (ORFs) [3]. Open reading frames 1a and 1b constitute 75% of the viral genome and encode viral replicase polyproteins, which are processed by self-encoded proteases into 14 non-structural proteins (nsp) [3]. Major (GP5, M, N) and minor (GP2, GP3, GP4, ORF5a-protein, E) structural proteins are being encoded at the 3’ end of the genome by ORFs 2a, 2b, 3, 4, 5, 5a, 6 and 7 [7]. Like with other nidoviruses, transcription of the PRRSV genome is characterized by the formation of a 3’-co-terminal nested set of subgenomic messenger RNAs, each of them with a common 5’-leader sequence [8].

Two distinct genotypes were identified, and designated as European type 1 (prototype LV-Lelystad, GenBank: M96262) and American type 2 (prototype VR2332, GenBank: AY150564), with 40% differences at the nucleotide level [7]. A large genetic variability has been shown within both subtypes [9]. Genetic heterogeneity is one of the main reasons why current commercial attenuated and inactivated vaccines provide incomplete protection against heterologous strains and as a result there is an urgent need for a novel generation of vaccines [10,11].

Starting from 2006, new highly pathogenic strains emerged, causing large-scale outbreaks in Eastern Europe (type 1, prototype Lena, GenBank: JF802085), and Southeastern Asia (type 2, prototype JXA1, GenBank: EF112445). These isolates are characterized by high viral loads in blood and tissues, high fever, severe general clinical signs and increased mortality [12-14]. A common genetic characteristic of highly pathogenic strains of both PRRSV type 1 and 2 is a discontinuous 30-amino acid deletion in the non-structural protein 2 (nsp2) (Lena: aa 710–739, JXA1: aa 534–563) [13,14]. However, this deletion is most probably not solely responsible for differences in virulence, as nsp2 is the most divergent one in PRRSV [7]. In Europe, highly virulent strains from Eastern Europe clearly differed from the circulating low virulent Western European strains and taxonomically they were designated as different type 1 subtypes (LV-like strains: subtype 1, Lena-like strains: subtype 3) [4]. Subtype 3 strains are constrained in Eastern Europe and up till now, there were no reports of highly pathogenic strains emerging in Western Europe [4].

In the present study, the virulence and pathogenicity of three PRRSV strains from Belgium were evaluated: two isolates (13V091 and 13V117) from 2013, originating from farms experiencing uncommon long-lasting anorexia, fever and respiratory problems within the first two weeks after weaning during an enzootic PRRSV infection, and the well-characterized Belgian strain 07V063 (GenBank: GU737264) from 2007 that was used as a reference [15,16]. Full-length genome sequencing was performed to characterize these PRRSV isolates.

## Materials and methods

### Viruses

PRRSV 13V091 and 13V117 were isolated from sera of pigs that showed severe respiratory problems in two Belgian farms. Virus isolation was performed as described before [12]. Briefly, porcine alveolar macrophages (PAM) were cultured in RPMI 1640 + GlutaMax modified medium (Gibco Invitrogen), supplemented with 10% fetal calf serum (FCS), 1% non-essential amino acids (Gibco Invitrogen), 1 mM sodium pyruvate and a mixture of antibiotics. Subsequently, cells were inoculated with ten-fold serial dilutions of sera and when cytopathic effect (CPE) was observed after three days, cells were fixed and stained with immunohistochemistry (IPMA) [10]. The monoclonal antibody (mAb) 13E2 against the nucleocapsid protein of PRRSV was used as primary antibody to identify PRRSV [17]. After isolation, 13V091 and 13V117 were grown in PAM and used for sequencing and pathogenesis studies. The viral stocks were confirmed to be free from Mycoplasma, porcine parvovirus, porcine circoviruses and swine influenza viruses. The 07V063 strain was enclosed as a reference. A 2^nd^-3^rd^ passage of 13V117, 13V091 and 07V063 was used for the animal inoculations.

### Experimental design

Thirty-six twelve-week-old pigs, originating from a PRRSV-negative farm, were randomly divided in 4 groups of 9 animals. Relevant pathogens (PRRSV, SIV, PCV2) were not detected in the animals. Experiments were conducted in a biosafety level 2 (BSL-2) facility and were approved by the Ethical Committee of the University of Ghent (EC 2010/090). The pigs of groups 13V091, 13V117 and 07V063 were inoculated intranasally (IN) with 2 mL containing 10^5^ tissue culture infectious dose with 50% end point (TCID_50_) of the respective viruses (1 mL per nostril). Group CON consisted of animals mock-inoculated with PBS. Rectal temperature and clinical observations were monitored daily, starting from the third day before challenge until the 21^st^ day post inoculation (dpi). Body temperature over 40.0 °C was considered as fever. The scoring system was used before and is fully described in previous studies [12,14]. Breathing, sneezing, coughing, nasal discharge, liveliness, ear discoloration, presence of peri-ocular oedema and diarrhea were taken into account. Detailed list and parameters of the clinical scores is shown in Additional file 1. In all groups, blood samples and nasal swabs were taken at 0, 3, 5, 7, 10, 14, 21, 28, 35 and 42 dpi. Plasma and nasal secretions were collected and stored at −70 °C for virus titration. At 10 dpi, 4 pigs from each group were euthanized and two samples were collected from the following tissues: nasal mucosa (septum and conchae), pharynx, tonsils, lymph nodes (pharyngeal, bronchial, mediastinal, inguinal), lungs (apical, cardiac and diaphragmatic lobes from both sides) and spleen. Lungs were examined macroscopically. One sample was embedded in methocel and was snap frozen for double immunofluorescense (IF) stainings, and the second sample was stored at −70 °C for virus titration.

### Gross pathology and bacteriology

After euthanasia, individual lungs were collected, and to all lung lobes macroscopic lesions were given a score to estimate the percentage of lungs affected by pneumonia using the evaluation system adapted from Halbur et al. [18]. Samples from lungs (cardiac left) and lymph nodes (bronchial) were collected after euthanasia and bacteriological analysis was performed. Each sample was inoculated on Columbia agar or Columbia CAN agar supplemented with 5% sheep blood (Oxoid, Hampshire, UK) with a *Staphylococcus pseudintermedius* streak for the growth support of NAD-dependent bacteria (*Actinobacillus, Haemophilus spp.*). Plates were incubated for 48 h in a 5% CO_2_-enriched environment at 35 ± 2 °C and phenotypic identification of isolated bacteria was performed as earlier described [19].

### Virus titration

Nasal secretions and 20% suspensions of the collected tissues were titrated on PAM as previously described [10]. Nasal swabs were collected using Aluminium Rayon sterile plain swabs (160C, Copan Italia S.p.A., Italy). After swabbing, the swabs were brought into transportation medium consisting of phosphate buffer saline (PBS), 10% fetal calf serum (FCS) and a mixture of antibiotics. Afterwards, the swabs were vortexed, and the diluted secretions were titrated [12]. Tissue suspensions were titrated on PAM in quadruplicate and a final TCID_50_ was determined after subjecting the cells to a PRRSV-specific immunoperoxidase staining to analyze the presence of PRRSV-positive cells.

### Serology

Serum samples were examined for the presence of PRRSV-specific antibodies using IPMA plates as described before [10]. In these plates, LV-infected MARC-145 cells were used to detect PRRSV-specific antibodies. Virus neutralizing antibody titers were detected with a seroneutralization (SN) test on MARC-145 cells after propagation of PRRSV strains on this cell line. Two fold dilution series of sera were made and an equal volume containing 100 TCID_50_ PRRSV of the homologous strains was added to each dilution and the mixture was further incubated for 1 h at 37 °C. Afterwards, the serum-virus mixture was transferred to suspension cultures of MARC-145 cells and the cells were analyzed for cytopathic effect (CPE) at ten days post inoculation. The virus neutralizing antibody titers were determined as the reciprocal of the highest dilution that inhibited CPE in 50% of the wells [16]. Samples that tested negative in IPMA were given a numerical value of 1.7 and in SN test a numerical value of 0.5.

### Quantification and identification of PRRSV-positive cells

To quantify and identify viral antigen and sialoadhesin (Sn) positive cells in the different tissues, several 9 μm cryosections were made at a distance of 50 μm between each other and fixed in 100% methanol at −20 °C for 15 min. Mouse monoclonal antibodies were used against the PRRSV N protein (13E2, 1:25, IgG_2a_) and porcine sialoadhesin (41D3, 1:2, IgG_1_) [18]. Isotype-specific secondary antibodies conjugated with FITC and Alexa Fluor 594 (1:500, Invitrogen) were used to reveal the different antigens. Cell nuclei were stained with Hoechst 33342 (Invitrogen) and to confirm the specificity of each antibody, negative isotype-specific control monoclonal antibodies were used: 13D12 against gD of PRV (IgG_1_), and 1C11 against gB of PrV (IgG_2a_) [20]. Countings were made in 5 sections with 5 fields per section measured in a blind way. Results were expressed per mm^2^, and analysis was performed using a Leica TCS SPE laser-scanning confocal microscope (Leica Microsystems GmbH, Wetzlar, Germany).

### Sequencing

Total RNA was extracted from 140 μL PAM culture supernatant of a 2^nd^ passage of 13V091 and a 3^rd^ passage of 13V117 strain using the QIAcube 230 volt robot and the QIAamp® Viral RNA Mini kit (QIAGEN, cat. No. 52906). The QIAamp Viral RNA body fluid standard program was used as the extraction protocol with the elution volume of 60 μL. The RNA was screened for PRRSV using the previously published Kleiboeker modified type 1 assay [21]. PCR products covering the whole PRRSV genome in two fragments were produced from full genome cDNA as described elsewhere [22]. The PCR fragments for each virus were pooled in equimolar concentration (165 ng in total) and sequenced by the Ion Torrent PGM sequencer using the 318v2 chip (DMAC, Technical University of Denmark). After initial removal of adaptors and low quality sequences, the quality of the output data (FastQ) files was examined using the applicant FastQC (v. 0.10.1). The reads were trimmed accordingly to the FastQC report. *De novo* assembly was performed using the commercial software CLC Genomics v. 4.6.1 (CLCBIO, Aarhus, Denmark) and the genetic comparability of the obtained contigs was found using the NCBI’s Basic Local Alignment Search Tool (BLASTn). The most similar sequence to the newly sequenced virus found by NCBI BLASTn was used as reference sequence for mapping reads to a reference. The final consensus sequence was found by alignment of contigs derived from *de novo* assembly and the sequence obtained from mapping to a reference. The consensus sequence from each virus was aligned to other publicly available full genome PRRS type 1 viruses using MUSCLE (Multiple Sequence Comparison by Log-Expectation). Gaps were either confirmed or closed by specifically designed primers surrounding the gaps and sequenced by cycle sequencing (LGC Genomics GmbH, Germany). All alignments and phylogenetic analysis, were performed by the commercial software CLC DNA workbench v. 6.9 (CLCBIO, Aarhus, Denmark). The phylogenetic trees were constructed using the Neighbor Joining algorithm, nucleotide/protein distance measure: Kimura80/Kimura Protein model, and Bootstrap analysis of 1000 replicates.

### Statistics

All data were analyzed with GraphPad Prism 6 software (GraphPad Software Inc., San Diego, CA, USA). Serological titers (IPMA and SN) as well as viral loads were log-transformed prior to the analyses. Gross pathology scores and area under the curve (AUC) were analysed using the non-parametric Kruskal-Wallis test with Dunn’s post-test. Statistical analysis of continuous data was performed using two-way analysis of variance (ANOVA) with Tukey’s post-test. All results shown represent means and standard deviation (SD). Results with *P*-values < 0.05 were considered significantly different.

## Results

### Clinical signs and body temperature

**Figure 1:**
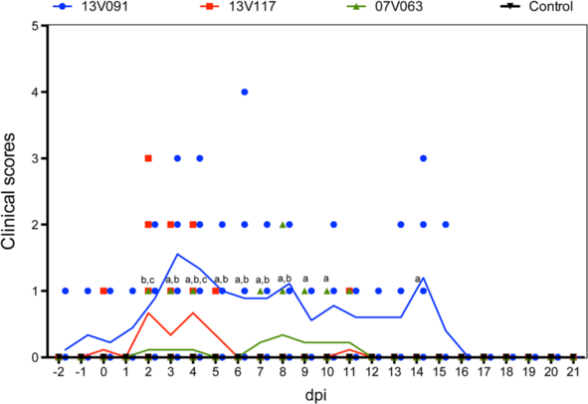
**Clinical scores in pigs inoculated with PRRSV 13V091, 13V117 and 07V063 are summarized.** Lines represent the mean value in each group. Letters denote significant statistical differences (*P* < 0.05) between viral strains (a: 13V091 and 13V117, b: 13V091 and 07V063, c: 13V117 and 07V063).

Clinical scores in respiratory functions, liveliness and presence of conjuctivitis are summarized in Figure 1. Daily registration of clinical scores for each individual animal is shown in Additional file 2. Animals from the control group did not display clinical signs during the whole study. The 13V091 group exhibited significantly (*P* < 0.05) higher mean scores from 3 till 10 and at 14 dpi compared to the 13V117 group, and from 2 till 8 dpi compared to the 07V063 group. All 13V091-infected animals scored positive for clinical signs at least at one time point during the experiment. Respiratory symptoms were observed from the beginning of the experiment with a peak at 4 dpi (8 out of 9 animals) and lasted until 15 dpi (1 out of 4 animals). The 13V117 group showed significantly (*P* < 0.05) higher mean scores compared to the 07V063 group at 2 and 4 dpi. Animals scored positive from 2 till 6 dpi but not all the animals showed respiratory disorders. A peak was observed at 4 dpi (4 out of 9 animals). In the 07V063 group, 2 animals showed clinical scores from 2 till 4 dpi and from 7 till 11 dpi. Loss of appetite was not observed in any group but visible growth retardation was marked in the 13V091 group compared to the other groups. Diarrhea was also apparent in the 13V091 group between 13 and 21 dpi. Nasal discharge, coughing or ear discoloration was never observed.

**Figure 2:**
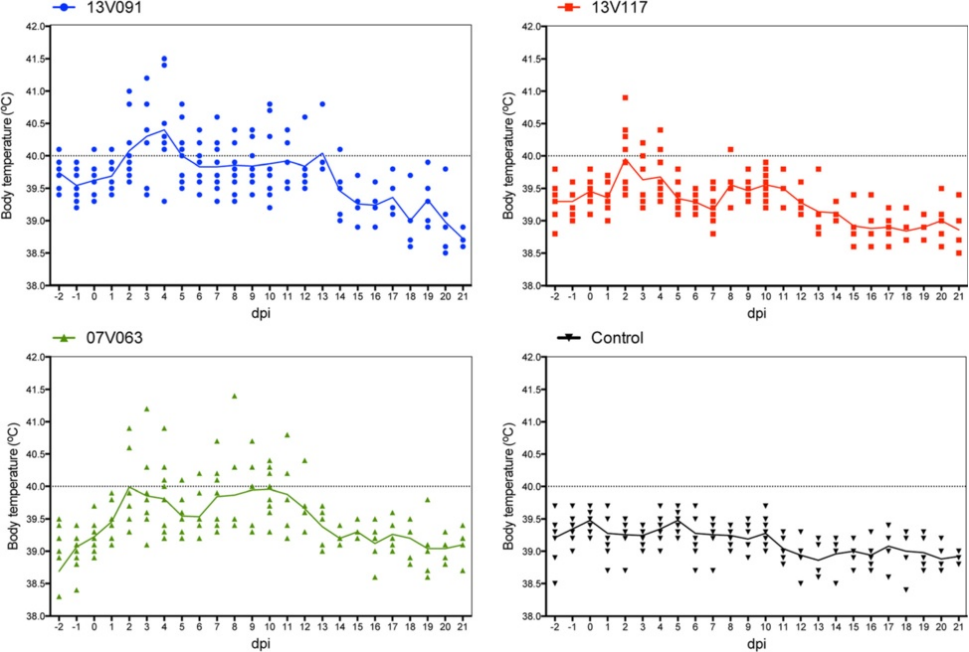
**Body temperature of pigs upon inoculation with PRRSV 13V091, 13V117 and 07V063.** Temperature ≥ 40 °C was considered as fever. Lines represent the mean value in each group.

Body temperature data are represented for each group in Figure 2. Detailed daily registration of body temperature for each individual animal is presented in Additional file 2. The mean body temperature of group 13V091 was significantly higher (*P* < 0.05) from 3 till 7 and at 13 dpi compared to the 13V117 group and from 3 till 5 dpi compared to the 07V063 group. In the 13V091 group, 8 out of 9 animals showed fever at least at one day during the experiment, whereas in the 13V117 and the 07V063 group, only 5 and 6 out of 9 animals showed fever, respectively. AUC values of individual pigs were calculated, and afterwards the AUC means per group were calculated and compared. The mean AUC value of increased body temperature with a threshold at 39.5 °C was significantly (*P* < 0.05) higher in the 13V091 group (6.0 ± 4.2) compared to the 13V117 (1.2 ± 1.3) and control groups (0.2 ± 1.3) but not to the 07V063 group (3.5 ± 3.4). 13V091-infected animals showed the highest mean number of fever days (5.1 ± 4.2 days) which was significantly different (*P* < 0.05) from the 13V117 group (1.1 ± 1.1 days) but not from the 07V063 group (3.4 ± 3.3 days).

### Gross pathology and bacteriology

**Figure 3:**
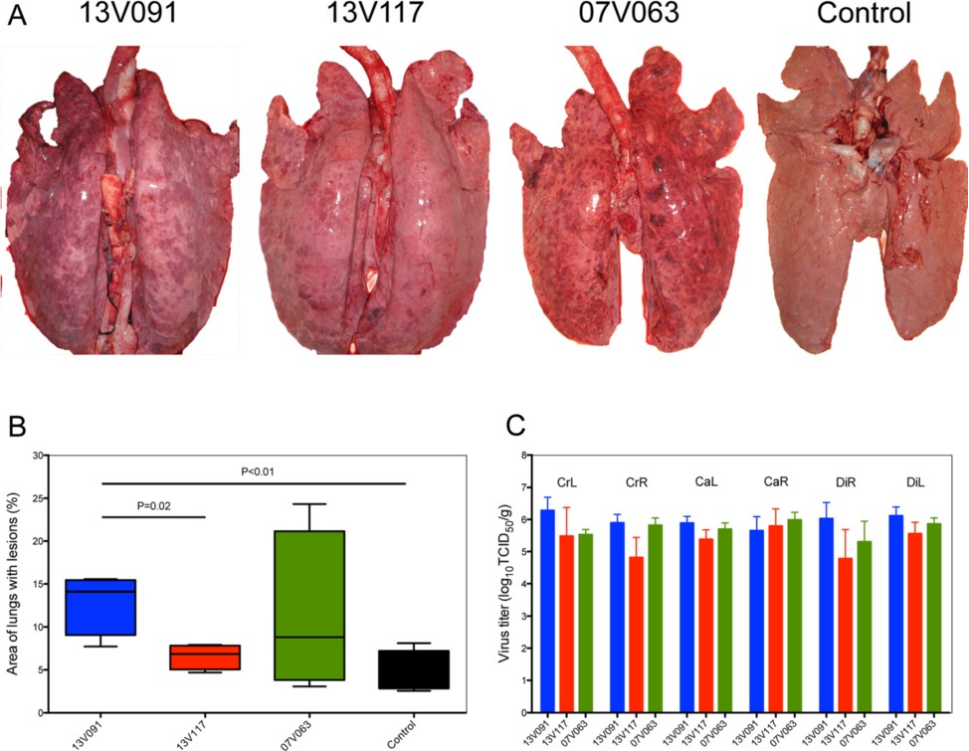
**Lung compartment analysis of animals infected with different PRRSV strains and euthanized at 10 dpi.** Representative gross pulmonary lesions **(A)**, mean macroscopic lesion scores **(B)** and virus titers in different lung compartments **(C)** are presented. CrL: Cranial Left, CrR: Cranial Right, CaL: Cardiac Left, CaR: Cardiac Right, DiL: Diaphragmatic Left, DiR: Diaphragmatic Right. Whiskers denote the standard deviation (SD) and *P* < 0.05 values represent statistically significant comparisons.

At 10 dpi, four pigs per group were euthanized and necropsied. Swollen pharyngeal, bronchial, mediastinal and inguinal lymph nodes were observed in the 13V091 and 07V063 infected animals. 13V091 induced a pneumonia that was characterized by tissue consolidation, haemorrhagic spots and multifocal red tanned lesions in both sides (Figure 3A). 13V117-infected animals showed also tissue consolidation and single spotted lesions located mainly at the cardiac lobe. In 07V063-infected pigs, tissue consolidation and multifocal bilateral red-tanned lesions, concentrated mainly at the cranial and cardiac lobes, were observed. No gross pulmonary lesions were found in negative control pigs, and no pleuritis and no Mycoplasma-related lesions were observed in all animals of the experiment. 13V091-infected pigs showed the highest mean percentage of lungs with gross lesions (12.9 ± 3.6%), which was significantly higher (*P* < 0.05) than the 13V117 group (6.5 ± 1.4%) and the control group (4.7 ± 2.4%) but was not different from the 07V063 group (11.3 ± 9.4%) (Figure 3B). Finally, samples from the cranial left part of the lungs and the bronchial lymph nodes of euthanized pigs were sent for bacteriological analysis. No specific bacterial pathogens were isolated.

### Virological analysis

**Figure 4:**
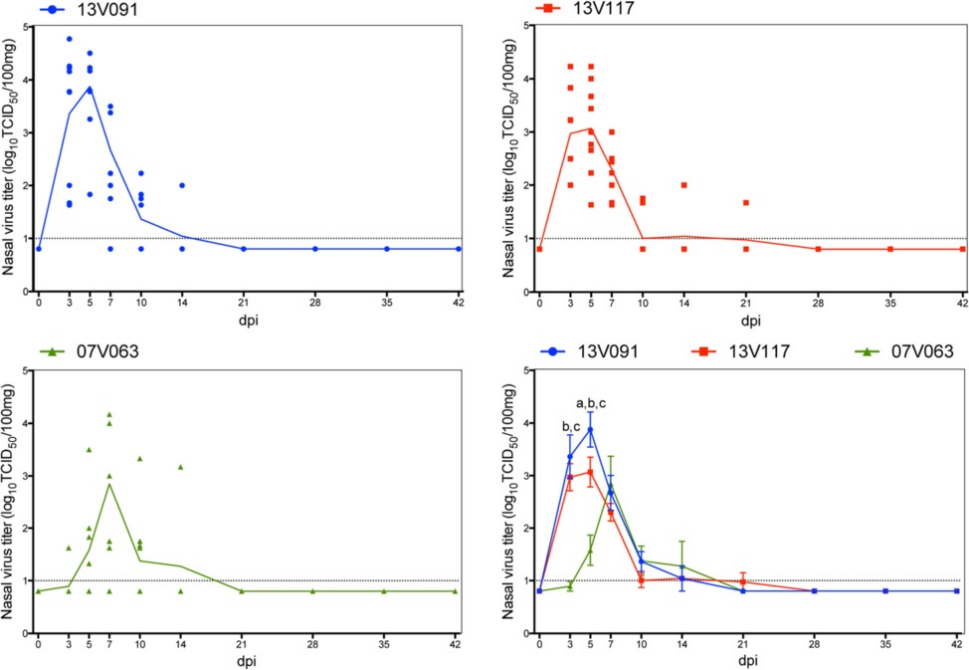
**Virus titers in nasal secretions of pigs inoculated with PRRSV 13V091, 13V117 and 07V063.** Lines represent the mean value in each group. Whiskers denote the standard deviation (SD). The dotted line gives the detection limit for virus titration. Letters denote significant statistical differences (*P* < 0.05) between viral strains (a: 13V091 and 13V117, b: 13V091 and 07V063, c: 13V117 and 07V063).

Titration of nasal swabs in the 13V091 group showed significantly higher mean titers (*P* < 0.05) at 5 dpi compared to the 13V117 group, and at 3 and 5 dpi compared to the 07V063 group. Group 13V117 exhibited significantly higher mean titers than group 07V063 at 3 and 5 dpi (Figure 4). The highest mean AUC value was observed in the 13V091 group (16.9 ± 3.7), which was significantly higher (*P* < 0.05) than the 13V117 (13.3 ± 4.3) and the 07V063 group (8.2 ± 4.3). Virus was detected for the first time at 3 dpi and two animals remained positive up till 14 dpi for the 13V091 and 07V063 groups, whereas in the 13V117 group one animal remained positive until 21 dpi. The 13V091 group showed the highest mean duration of nasal shedding (5.8 ± 2.4 days), which was not significantly different from the nasal shedding duration of the 13V117 (5.4 ± 2.4 days) and 07V063 (3.3 ± 1.9 days) group.

**Figure 5:**
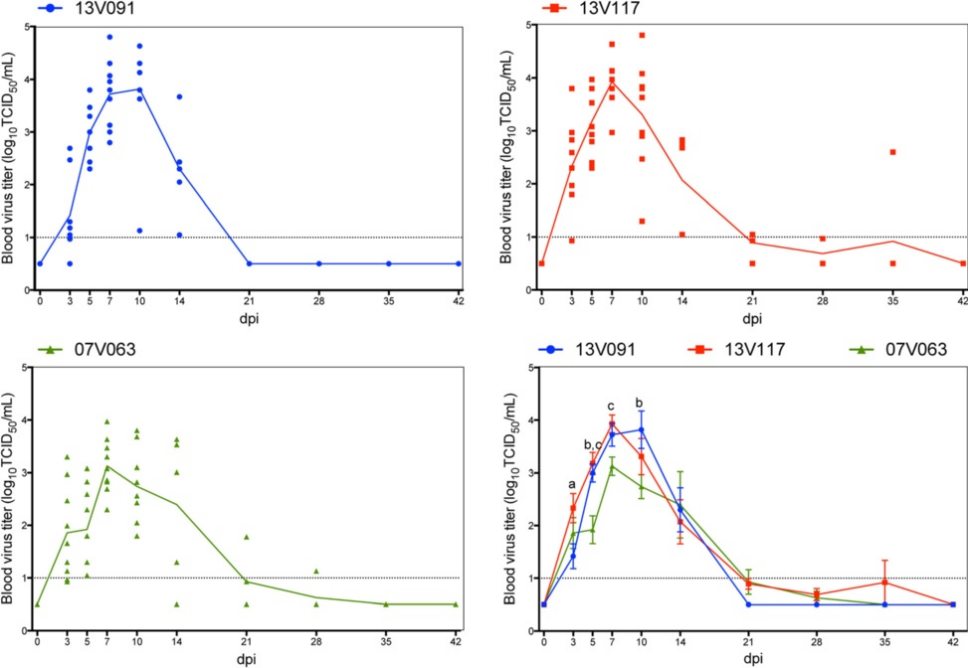
**Viremia in pigs inoculated with PRRSV 13V091, 13V117 and 07V063.** Patterns represent individual animals. Lines represent the mean value. Whiskers denote the standard deviation (SD). The dotted line gives the detection limit for the virus titration. Letters denote significant statistical differences (*P* < 0.05) between viral strains (a: 13V091 and 13V117, b: 13V091 and 07V063, c: 13V117 and 07V063).

Viremia was first detected at 3 dpi and lasted until 14 dpi for the 13V091 group. Two pigs in the 13V117 and 07V063 groups remained positive until the 35^th^ and 28^th^ dpi, respectively. The 13V091 group showed significantly higher (*P* < 0.05) mean virus titers at 5 dpi compared to the 13V117 group, and at 5 and 10 dpi compared to the 07V063 group. The 13V117 group exhibited significantly higher mean titers at 5 and 7 dpi compared to the 07V063 group (Figure 5). Control animals did not show viremia during the experiment. 13V091 showed the highest duration (8.8 ± 1.9 days) compared to 13V117 (8.1 ± 1.8 days) and 07V063 (6.9 ± 0.7 days). The average AUC value of the 13V091 (22.2 ± 9.3) and the 13V117 group (25.1 ± 8.4), were slightly higher than that of the 07V063 group (18.7 ± 10.5) (*P* > 0.05).

### Serology

**Figure 6:**
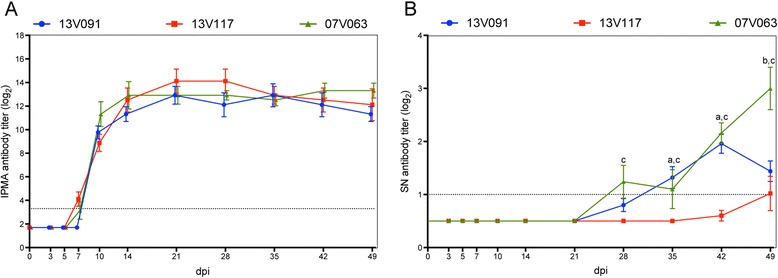
**Antibody response, IPMA (A) and SN (B) in pigs inoculated with PRRSV 13V091, 13V117 and 07V063.** Whiskers denote the standard deviation (SD). Dotted lines denote detection limits. Letters denote significant statistical differences (*P* < 0.05) between viral strains (a: 13V091 and 13V117, b: 13V091 and 07V063, c: 13V117 and 07V063).

PRRSV-specific antibodies were first detected with IPMA in all PRRSV-infected groups as early as 7 dpi (Figure 6A). The highest mean antibody titers ranged from 2^12.9^ to 2^14.1^ for all groups and were reached at 21–35 dpi. No significant difference was observed between the infected groups at any time point. Control animals remained seronegative during the whole experiment.

In 07V063-infected animals, homologous SN titers appeared at 28 dpi in three out of five pigs, and reached a maximum mean titer at 49 dpi (2^3^) when all animals were positive. In the 13V091 group, SN titers against the homologous strain could be detected in three pigs at 28 dpi (2^1^) and the titers remained at a similar level until 49 dpi when all the animals became positive and the group showed a mean titer of 2^1.4^ ± 2^0.4^ (Figure 6B). 13V117-infected animals did not show any SN titer against the homologous strain until 42 dpi when one pig out of five was positive (2^1^). At 49 dpi, one additional pig became positive (2^2^).

### PRRSV titers in different organs

#### Respiratory tract

**Figure 7:**
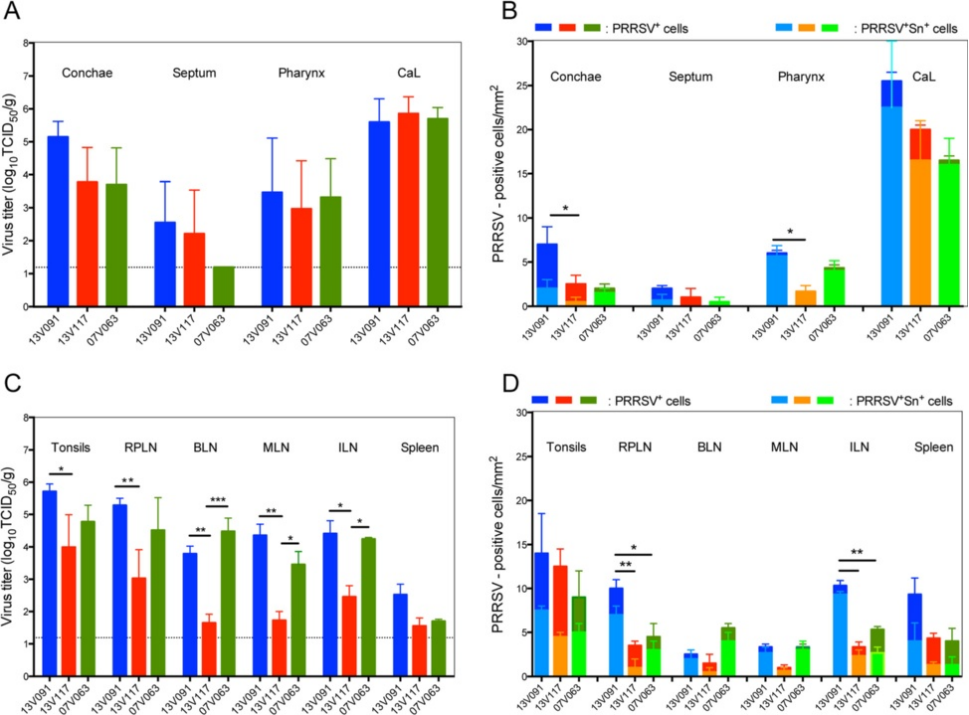
**Virus titers (A and C), and quantification and identification of PRRSV-infected cells (B and D).** Different parts of the respiratory system and lymphoid tissues from animals inoculated with PRRSV 13V091, 13V117 and 07V063, and euthanized at 10 dpi were analyzed. Dark colors represent PRRSV^+^ cells, whereas lighter colors in the subcolumns represent PRRSV^+^Sn^+^ cells. CaL denotes the cardiac left part of the lungs. Whiskers denote the standard deviation (SD) and asteriscs (*) represent statistically significant comparisons (*P < 0.05, **P < 0.01, ***P < 0.001).

Tissues from animals euthanized at 10 dpi were collected for PRRSV titration. In the conchae, 13V091-infected animals showed higher (*P* > 0.05) mean virus titers (5.1 ± 0.9 log_10_TCID_50_/g) than 13V117 (3.8 ± 2.1 log_10_TCID_50_/g) and 07V063 infected animals (3.7 ± 2.2 log_10_TCID_50_/g) (Figure 7A). In the 13V091-infected animals, septum and pharynx displayed titers of 2.6 ± 1.2 and 3.5 ± 1.6 log_10_TCID_50_/g respectively, which were slightly higher than the ones in the 13V117 group (septum: 2.2 ± 1.3 log_10_TCID_50_/g, pharynx: 3.0 ± 1.4 log_10_TCID_50_/g) and the 07V063 group (septum: 1.2 ± 0.1 log_10_TCID_50_/g, pharynx: 3.3 ± 1.1 log_10_TCID_50_/g), but not significantly different. Slightly higher titers for the 13V091 group were found after titration of lung compartments (Figure 3C), and values from all strains ranged from 4.8 to 6.2 log_10_TCID_50_/g. Samples from the pigs of the control group were negative for virus isolation.

#### Lymphoid tissues

Average viral titers in tonsils (5.7 ± 0.5 log_10_TCID_50_/g) and retropharyngeal lymph nodes (RPLN) (5.2 ± 0.4 log_10_TCID_50_/g) in the 13V091 group, were significantly higher (*P* < 0.05), than in the 13V117 group (tonsils: 4.0 ± 2.0 log_10_TCID_50_/g, RPLN: 3.0 ± 1.8 log_10_TCID_50_/g), but not from the 07V063 group (tonsils: 4.8 ± 1.0 log_10_TCID_50_/g, RPLN: 4.5 ± 2.0 log_10_TCID_50_/g). Virus titration of bronchial (BLN), mediastinal (MLN) and inguinal (ILN) lymph nodes of the 13V091 (BLN: 3.8 ± 0.4 log_10_TCID_50_/g, MLN: 4.4 ± 0.7 log_10_TCID_50_/g, ILN: 4.4 ± 0.8 log_10_TCID_50_/g) and 07V063 groups (BLN: 4.5 ± 0.8 log_10_TCID_50_/g, MLN: 3.4 ± 0.8 log_10_TCID_50_/g, ILN: 4.2 ± 0.1 log_10_TCID_50_/g), showed significantly (*P* < 0.05) higher titers compared to the 13V117 group (BLN: 1.6 ± 0.5 log_10_TCID_50_/g, MLN: 1.7 ± 0.5 log_10_TCID_50_/g, ILN: 2.5 ± 0.7 log_10_TCID_50_/g) (Figure 7C). Finally, in spleen, the 13V091 group showed the highest titers (2.5 ± 0.6 log_10_ TCID_50_/g) of the PRRSV-infected groups (*P* > 0.05).

### Immunofluorescence

#### Respiratory tract

Double IF stainings were performed for the quantification and identification of PRRSV cells. In conchae, 13V091 showed higher amounts of PRRSV^+^ cells/mm^2^ (8.0 ± 1.4) compared to 13V117 (2.5 ± 0.7) and 07V063 (4.3 ± 0.7) (Figure 7B). Tissues from the septum displayed also a higher amount of PRRSV^+^ cells/mm^2^ in the 13V091 (2.2 ± 1.4) group, compared to the 13V117 (0.8 ± 0.9) and 07V063 (0.6 ± 0.3) groups. In the pharynx, a higher (*P* < 0.05) number of PRRSV^+^ cells/mm^2^ (6.0 ± 1.7) was found with 13V091 than with 13V117 (1.7 ± 1.2) but not from 07V063 (4.3 ± 1.2). Finally, immunofluorescence stainings from the left cardiac lobe of the lung showed higher amounts of PRRSV^+^ cells/mm^2^ for 13V091 (25.5 ± 9.1) compared to 13V117 (20.3 ± 5.4) and 07V063 (16.5 ± 2.3). The number of infectious virus particles per infected cell in the left cardiac lung lobe was calculated according to the ratio: pfu per gram of tissue/cells per mm^2^ after converting TCID_50_/g to pfu/g [23]. 07V063 showed the highest number (21 200) of infectious virus particles per infected cell compared to 13V091 (15 300) and 13V117 (8600).

**Figure 8:**
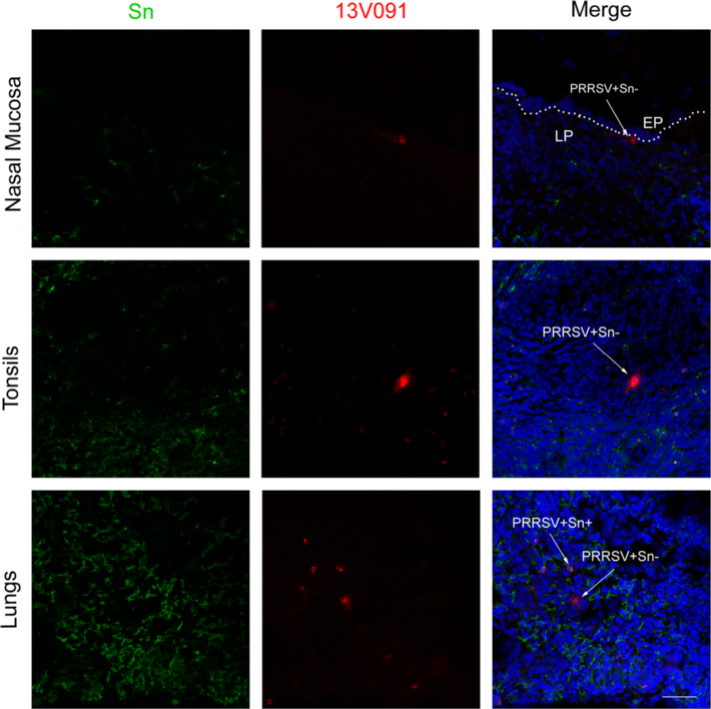
**Confocal microscope double immunofluorescence images of 13V091-infected nasal mucosa, tonsils and lungs are illustrated.** Tissue samples were sectioned (9 μm) and co-immunostained for Sn (green) and PRRSV N-protein (red) at 10 dpi. White arrows show PRRSV^+^Sn^+^ and PRRSV^+^Sn^−^ cells. Scale bar 50 μm.

The Sn phenotype of PRRSV^+^ cells in the nasal mucosa, tonsils and lungs is shown in Figure 8. Identification of PRRSV^+^ cells in the nasal mucosa based on Sn, revealed that the 13V091 and 13V117 strains were able to replicate more efficiently in Sn^−^ cells compared to the 07V063 strain. In conchae, 87.5% of 13V091-infected cells were Sn^−^, while that percentage was 80.0% with the 13V117 and only 17% with the 07V063 strain (Figure 7B). In the septum, half (48%) of the 13V091-infected cells and the few 13V117-infected cells (0.8 ± 0.9/mm^2^) were Sn^−^, whereas all 07V063-infected cells were Sn^+^. In the pharynx and lungs, all isolates showed a restricted cell tropism, as the percentage of PRRSV^+^Sn^+^ cells ranged from 80.7% to 100% (Figure 7B).

#### Lymphoid tissues

In tonsils, a higher amount of PRRSV^+^ cells/mm^2^ was found in 13V091-infected tissues (14.0 ± 5.6), compared to 13V117 (12.5 ± 2.1) and 07V063 (9.0 ± 5.7) -infected tissues (Figure 7D). In BLN, a higher amount of PRRSV^+^ cells/mm^2^ was observed in the 07V063 group (5.5 ± 2.2) than in the 13V091 (2.6 ± 0.8) and 13V117 (1.5 ± 0.7) groups, whereas in RPLN, 10.0 ± 2.8 (13V091), 2.5 ± 0.6 (13V117) and 4.5 ± 3.5 (07V063) PRRSV^+^ cells/mm^2^ were found. Finally, in MLN, infection with the 13V091 and 07V063 strains resulted in 3.4 ± 1.5 and 3.3 ± 2.3 PRRSV^+^ cells/mm^2^ respectively, while infection with the 13V117 strain gave only 1.1 ± 0.8 PRRSV^+^ cells/mm^2^. The amount of PRRSV^+^ cells in ILN was higher (*P* < 0.05) with the 13V091 strain (10.3 ± 0.5) compared to the 13V117 (3.3 ± 2.1) and 07V063 (5.3 ± 0.6) strains. In spleen, the highest amount of PRRSV^+^ cells/mm^2^ was displayed by 13V091 (9.3 ± 6.7) while 13V117 showed 4.3 ± 3.5 and 07V063 4.0 ± 2.8 PRRSV^+^ cells/mm^2^.

The highest percentage of infected cells that were Sn^−^, ranged from 45% to 64% in tonsils, 20% to 68% in the different lymph nodes and 64% to 71% in the spleen.

### PRRSV genome sequencing and phylogenetic analysis

The full genomes of 13V091 and 13V117 were 15020 and 15014 nucleotides (nt) long (excluding the polyA tail), respectively. A NCBI BLASTn search showed that the two viruses belong to the PRRSV type 1 genotype. Pairwise nucleotide comparisons of the complete genomes showed that 13V117 shared 91.55% identity to Lelystad virus and 99.81% identity to 07V063, whereas 13V091 only shared 86.69% identity to the Lelystad virus and 85.03% to the 07V063 strain. The nucleotide (nt) similarity between 13V091 and 13V117 was 84.38%. 13V091, 13V117 and 07V063 showed deletions in ORF1a, in the region encoding the non-structural protein 2 (nsp2). 13V117 had a 84 nt deletion following the nucleotide position 2345 in LV. 13V091 had three discontinuous deletions of 3, 45, and 27 nucleotides following the nt positions 1919, 2384, and 2492 in LV, respectively. The first deletion of 3 nt has also been found in five strains isolated in Asia (GenBank: FJ349261, KF287128-31). The deletions of 45 and 27 nucleotides were located in the highly variable region of nsp2 close to the deletion seen in 13V117. A partial amino acid alignment representing the highly variable region of nsp2 is shown in Additional file 3. The 13V091 strain, also harbored a 3 nt deletion in the region of the genome encoding the structural proteins. This deletion affected both ORF3 and ORF4 as the coding regions of these two genes overlap, but did not disturb the open reading frame [24].

**Figure 9:**
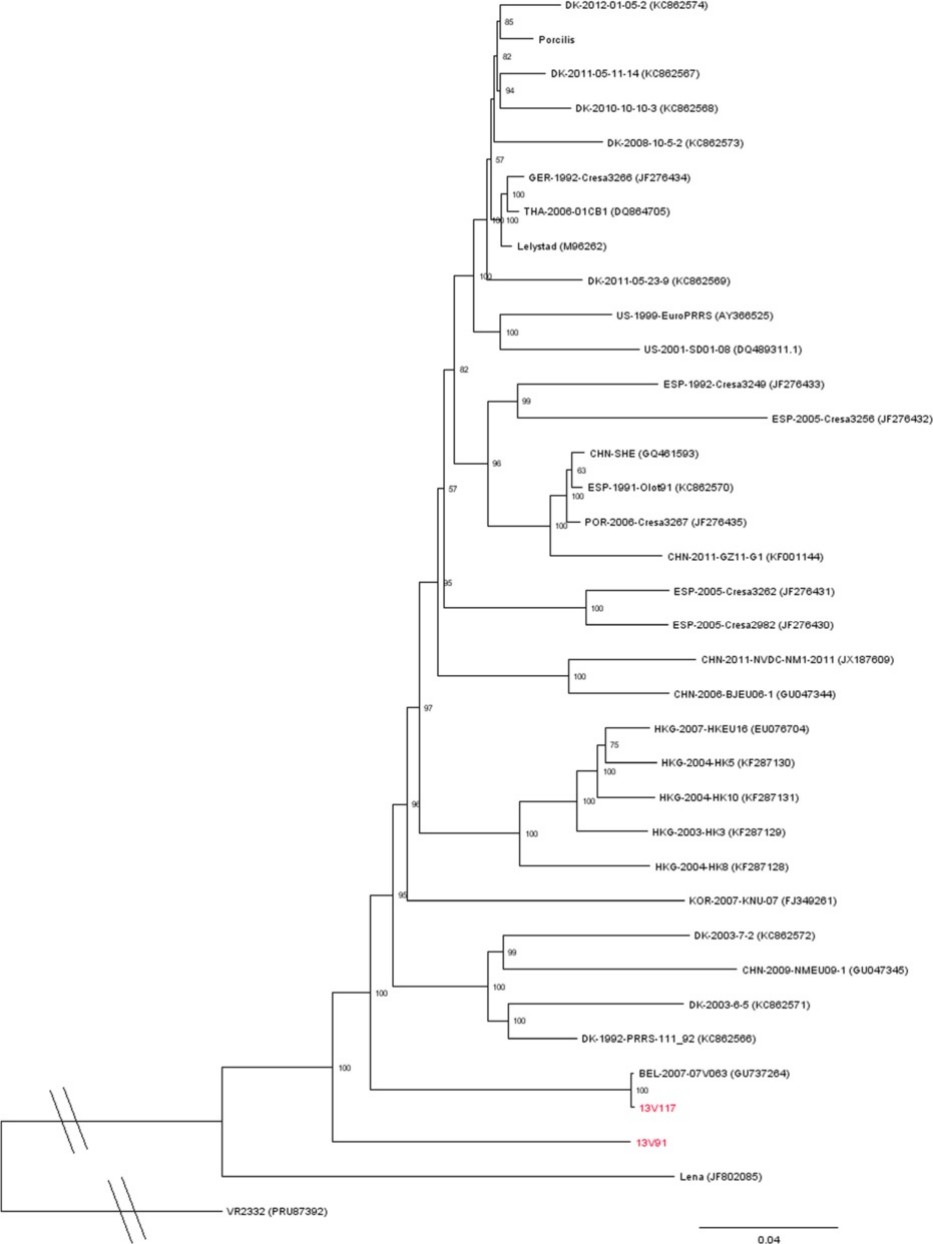
**Phylogenetic analysis of complete genome nucleotide sequences of PRRSV type 1 is presented.** The tree was constructed by the Neighbor Joining algorithm with Bootstrap 1000 replicates. Bootstrap values are shown in percent. 13V091 and 13V117 are highlighted in red.

Phylogenetic analysis of the complete genomes of the two viruses to 33 published PRRSV type 1 sequences showed that 13V091 grouped alone, and 13V117 grouped with 07V063 (Figure 9). Phylogenetic analysis of 171 complete PRRSV type 1 ORF5 sequences showed that 13V117 grouped with 07V063 and with viruses isolated in Denmark, China, Korea, and Spain, whereas 13V091 grouped with viruses isolated in Romania. The phylogenetic analysis of ORF5 is presented in Additional file 4. The phylogenetic tree of ORF7 was constructed from 154 sequences and showed 13V117 to group with 07V063 and viruses from Hong Kong, Korea, and Thailand. 13V091 grouped with an Italian isolated virus.

To investigate the impact of the high genetic diversity of 13V091 and 13V117, a pairwise amino acid comparison of the case viruses to three other type 1 viruses was performed and is shown in Additional file 5. From the comparison, it was clear that both 13V091 and 13V117 showed a high level of diversity on the protein level to the LV virus, the subtype 1 prototype and to Lena, the subtype 3 prototype virus. 13V091 was also different from 13V117 and 07V063, where the percentage identity for nsp2, GP3, GP4 and GP5 ranged from 77% to 88%. Finally, full-genome analysis revealed that 13V117 and 07V063 only differed by 8 amino acids located in nsp1, nsp2, nsp7 nsp9, GP3 and GP4, respectively.

## Discussion

PRRSV is one of the most economically important pathogens in swine industry worldwide [2]. The virus continues to evolve genetically and antigenically despite the efforts for control and eradication [7]. Genetically, PRRSV strains are divided into European type 1 and North-American type 2 strains [8]. Type 1 strains are subdivided in four subtypes (1, 2, 3 and 4). In the recent past, highly pathogenic type 1 subtype 3 strains emerged in Eastern Europe but there was no indication of an invasion of these strains in Western Europe, a region that is dominated by type 1 subtype 1 isolates [4]. Up till now, no highly pathogenic type 1 subtype 1 strains have been isolated. Continuous monitoring of emerging PRRSV isolates is of great importance in order to prevent new outbreaks and provide novel insights for vaccine development. In the current study, the pathogenicity and the virulence of two new Belgian PRRSV isolates designated 13V091 and 13V117 originating from animals experiencing severe respiratory disorders were compared clinically, virologically and genetically with the mild-pathogenic PRRSV strain 07V063.

PRRSV 13V091 appeared to be more pathogenic than the 13V117 and 07V063 strains. Animals infected with 13V091 showed the longest duration of fever (5.1 ± 4.2) and the highest clinical scores (1.6 ± 0.3) due to dyspnea, tachypnea and respiratory distress. Macroscopic analysis of lungs of pigs euthanized at 10 dpi showed that 13V091 strain caused the most severe multifocal gross lesions compared to the 13V117 and 07V063 isolates. Percentage of lung lesions of 13V091 was also higher than that of the type 1 strains that were used in a recent study from Spain [25]. In another Korean study, three new subtype 1 strains were compared with the prototype LV, and it was found that at 10 dpi only one of them (SNUVR100744, GenBank: JX988618) showed a higher percentage (>25%) of affected lung surface than 13V091 (12.9 ± 3.6) [26]. The clinical and pathological outcome of 13V091 showed some similarities with the highly pathogenic subtype 3 strain (Lena, GenBank: JF802085) [12,14]. In the latter study, Lena showed clinical score values ranging from 0.5 to 2 from 3 to 14 dpi, whereas in our results 13V091 showed a clinical score from 0.6 to 1.6 during the same timeframe. Previous in vivo studies with the Lena strain, induced fever from 2 till 13 dpi [14], and from 2 till 28 dpi [12]. In the current study, 13V091 induced fever from 2 till 14 dpi.

The clinical outcome can be fully attributed to PRRSV and not to PRRSV-related pathogens (SIV, PCV2). Experiments were performed in BSL-2 facilities and pathogenic bacteria were not isolated from all the euthanized pigs. Furthermore, no Mycoplasma-related lung lesions were observed in all animals. Co-infections are important because they enhance the impact of PRRSV leading to porcine respiratory disease complex (PRDC) [27]. In a previous study with highly pathogenic PRRSV strains that showed mortality, bacteria like *Arcanobacterium pyogenes* and *Streptococcus suis* were isolated from the lungs [12].

Transmission of PRRSV via airborne route is an important characteristic after the appearance of highly pathogenic viral strains [28]. In a previous study, virus titers in nasal secretions of a highly pathogenic strain (Lena) showed a titer of 2.5 to 5.6 log_10_TCID_50_/100 mg from 3 to 14 dpi [12]. In the current study, during the same timeframe, viral titers of 2.0 to 4.8 and 1.8 to 4.3 log_10_TCID_50_/100 mg were detected for 13V091 and 13V117 respectively. These results indicate that both new strains although not as pathogenic as Lena, have the potential to be transmitted via airborne route in the early stages of infection based on the high virus titers in the nasal secretions. It has been speculated, that recent outbreaks in the USA and China were due to virus isolates that can replicate and spread more efficiently [29]. The location of virus replication and an expanded cell tropism may strongly support this hypothesis. In typical swine herds, animals are in close nose-to-nose contact with each other, and during the last years several studies were performed evaluating the efficiency of different isolates to spread via airborne route [30]. A PRRSV strain that is able to replicate in the nasal mucosa and produce progeny virus, will be more likely to spread to another pig via airborne route compared to a virus strain that mainly replicates the lungs such as typical LV-like strains. In addition, presence of other nose pathogens such as *Bordetella bronchiseptica* and *Chlamydia suis* may cause intensive sneezing, which may facilitate virus spread [31].

In this study, different PRRSV strains showed a different tissue tropism. Animals infected with the 13V091 strain, showed the highest replication efficiency based on virus titration and PRRSV-positive cell counting. Furthermore, 13V091 showed significantly higher numbers of PRRSV^+^ cells compared to 13V117 and 07V063 in the upper respiratory tract, tonsils and draining lymph nodes (conchae, septum, pharynx, RPLN). Respective virus titers of 13V091 were 10 to 1000 times higher than 13V117, and 10 times higher than 07V063. In the upper respiratory tract, 13V091 and 13V117 showed an expanded cell tropism as they were shown to replicate in Sn^−^ macrophages with a different degree of efficiency. It is important to mention that in the nasal mucosa (conchae, septum), the same distribution pattern of PRRSV^+^Sn^−^ cells was observed for 13V091 and 13V117, with cells being located within the epithelium, and just underneath the basement membrane. Large round cells with large cytoplasm was the morphology of PRRSV^+^Sn^−^ and PRRSV^+^Sn^+^ cells located in the lamina propria, whereas PRRSV^+^Sn^−^ cells located within the epithelium and just underneath the basement membrane were smaller with large elongated filopodia. Together with the disability of Lelystad virus to grow in Sn^−^ cells and the strong ability of a highly pathogenic PRRSV strain (Lena) to replicate extremely efficiently in Sn^−^ cells of the nasal mucosa, this indicates that in Western Europe there is an increase of PRRSV tropism for Sn^−^ nasal macrophages in time, which reaches the level of virulent/pathogenic subtype 3 strains [32].

In our study, 13V091 showed a higher replication rate and produced more progeny virus in the lungs compared to 13V117 and 07V063. Viral loads of type 1 subtype 1 strains of our study in lungs, are in agreement with the results of Labarque et al., where LV reached a mean titer of 10^6.2^ TCID_50_/g in lung tissues at 9 dpi [10]. It was shown before, that PRRSV replication in the lungs is correlated with the number of the susceptible Sn^+^ macrophages that are present at the time of infection and that only 2% of susceptible macrophages are infected [10]. In the present study, 13V117 and 07V063 showed a similar number of viral antigen positive cells and virus titers in the left cardiac lung lobe (13V117: 20.3 cells/mm^2^ and 10^5.4^ TCID_50_/g, 07V063: 16.5 cells/mm^2^ and 10^5.7^ TCID_50_/g). Analysis of the virus production per infected cell showed that 13V091 and 07V063 were able to produce more infectious virus particles per infected cell than 13V117 in a ratio of 1:2 and 1:2.4 respectively. Thus, not only the number of susceptible cells, but also the strain-dependent replication efficiency is important for PRRSV lung pathogenesis. Differences in replication level within distinct lung lobes were not observed with all the tested strains, which correlate with two other studies with the Lelystad (LV) virus, where no difference in replication levels of distinct lung lobes was observed at 10 dpi [10,18].

Virus replication in BLN and MLN showed that 13V091 and 07V063 produced similar amounts of virus, which were up to 1000 times higher than 13V117. High viral loads of 13V091 and 07V063 in the lungs and the draining lymph nodes (BLN, MLN) can be explained by the mobilization of Sn^+^ cells from lungs to LN. In addition, strains with an expanded cell tropism like 13V091, may use the Sn^−^ monocytic cells that are infiltrating after 9 dpi to increase their transportation and replication efficiency in the correspondent tissues [10].

In the spleen, 13V091 showed the highest replication level compared to the other tested isolates and the virus production reached 10^2.8^ TCID_50_/g at 10 dpi, which was similar to LV (10^3^ TCID_50_/g) at 8 dpi [33]. More than 60% of PRRSV-infected cells were of Sn^−^ phenotype. The lower replication level observed in spleen compared to lungs and lymph nodes might be explained by the low expression of Sn and presence of other receptor(s). In rats, it was shown that Sn expression on splenic macrophages is reduced 25-fold compared to lymph node macrophages mainly due to masking of Sn by endogenous ligands [34].

In our study, PRRSV-specific antibodies for all strains appeared at 7 dpi and reached a plateau at 14 dpi, which is in agreement with previous studies using other PRRSV strains [10,35]. The late induction and the low titers of neutralizing antibodies observed in the current study is in agreement with other studies that used the LV and the 07V063 strains, where only low titers of Nabs were observed after 25 dpi for LV (2^1–3.6^), and after 35 dpi for 07V063 (2^1–2.2^) [10,16]. A correlation between the appearance of neutralizing antibodies (Nabs) and the virus elimination from blood was mentioned before, but the exact degree of Nabs contribution in viral clearance is still questionable [35-37]. Only in the 07V063 group Nabs appeared at the same time with the elimination of viremia, an observation that was reported before for the 07V063 strain [16]. In 13V091 and 13V117, Nabs appeared later and at lower titers, and at that time the virus was already eliminated from blood. Based on these observations, it is obvious that neutralizing antibodies are not the sole immunological tool to eliminate PRRSV, and their role in clearance of primary viremia and prevention of reinfection is not clear [38]. The presence of additional glycosylation sites at the neutralizing epitopes, and the sensitivity of the SN test, might contribute for the late development of Nabs observed in 13V091 and 13V117 compared to 07V063. Analysis of glycosylation sites on PRRSV structural proteins based on Lelystad virus, revealed one additional glycosylation site for 13V091 into GP3 protein at the amino acid position 253 [39]. No additional or different glycosylation sites were observed within 13V117 and 07V063. Addition of newly acquired sugar moieties to the sugar tree of 13V117 may make the virus unrecognizable and mask the neutralizing epitopes that reduce or eliminate the binding affinity of the antibodies. This mechanism of immune evasion has been well described for HIV-1 [40]. MARC-145 cells and PRRSV strains adapted on this cell line are generally used in SN tests. This cell type is not the natural cell-target for PPRSV and does not express Sn, and CD163 is not present at detectable levels. Both Sn and CD163 are main factors for PRRSV entry and replicaton, and are major targets for viral entry mediators (GP5-M for Sn and GP2-GP3-GP4 for CD163) [41,42]. Therefore, primary target cells like alveolar macrophages or cell lines that express Sn and CD163 like PK15^Sn-CD163^ cells should be preferably used for all the contemporary PRRSV strains that have a main tropism for PAM [43]. Newly emerging isolates with a tropism for additional receptors might need another SN test based on primary monocytic cells from nasal mucosa or lymph nodes that express the new receptors to search for neutralizing antibodies. New SN tests might help to better understand the function of virus Nabs, with regard to the degree of viral clearance.

To study the evolutionary relationship of 13V091 and 13V117 with other PRRSV strains, the complete genome sequence was compared with other type 1 European strains and the prototype type 2 strain VR-2332. 13V091 showed a high genetic diversity to all, currently, publicly available complete genomes of type 1 viruses and was located “alone” in the phylogenetic tree between 13V117 and 07V063, and the highly pathogenic subtype 3 strain Lena. The phylogenetic analysis of ORF5 and ORF7 showed that 13V091 and 13V117 grouped with other type 1 subtype 1 sequences, thus, it can be concluded that both isolates belong to PRRSV type 1 subtype 1.

By coincidence, 13V117 showed an extremely high genetic similarity to 07V063. Despite the fact that genome comparison revealed only eight different amino acids, the virological, clinical and pathological changes induced by these two viruses were quite different. 13V117 replicated efficiently in the nasal mucosa, induced a high viremia but had problems to replicate in lymphoid tissues. In addition, 13V117-infected animals showed breathing problems between 2 and 7 dpi. On the other hand, animals from the 07V063 group, showed a restricted replication in the nasal mucosa, a ten-fold lower viremia but an extensive replication in the lymphoid tissues. Only two pigs had mild breathing problems. Pairwise alignments revealed that the following eight amino acid differences were observed between 07V063 and 13V117: V140M in nsp1, H786L and G1300S in nsp2, S2192N in nsp7a, M72I and M266V in nsp9, N253H in GP3 and Q72P in GP4. Nsp1 is a cysteine protease that plays an important role in the cleavage of ORF1 proteins and for virion biogenesis [44]. Nsp2 is the papain-like proteinase 2 (PLP2) that is encoded from ORF1a and is responsible for the cleavage of the nsp2-3 junction [7]. It is the most variable non-structural protein between PRRSV subtypes, and it contains a hypervariable region (HVR) in which deletions are commonly found among PPRSV isolates; a cluster of immunodominant B- and T-cell epitopes was also identified [7]. Deletions in nsp2 have also been found for 13V091 and 13V117. Recent data support the hypothesis that these deletions are not necessarily related to virulence, but other studies support that changes in the nsp2 region might be related with plaque appearance and the cytolytic activity of the isolate [45,46]. Nsp7a is a 149 aa protein that plays an important role in the induction of host humoral immune response and has been found to induce specific antibodies as early as 14 dpi till 202 dpi [47]. Nsp9 is the RNA-dependent RNA polymerase (RdRp) encoded by ORF1b. It mediates the genome replication and the synthesis of subgenomic mRNAs, and is one of the most conserved proteins within nidoviruses [7]. GP3 is a heavily glycosylated membrane protein with a hydrophobic C-terminal domain [39]. The single amino-acid change in GP3 between 07V063 and 13V117 is located within the neutralizing antigenic region in the C-terminal domain, that has been found to be recognised by neutralizing antibodies against LV [39]. GP4 is a membrane protein with an N-glycosylated ectodomain that forms a heterotrimer with GP2 and GP3, and interacts with GP5 [42]. The single amino acid difference between 07V063 and 13V117 is located at the ectodomain of GP4 within the most immunogenic region that was identified, in all envelope proteins, but is also part of a neutralizing antibody region (NAR) named ES12 found to induce high virus neutralizing antibody titers at 42 dpi in all the 6 tested animals of the study [42,48]. In our results, only one animal in the 13V117 group showed low virus neutralizing antibody titers at 42 dpi, whereas at the same timepoint all animals in the 07V063 group developed virus neutralizing antibody titers (mean titer: 2^2.16^). Whether these single amino acid substitutions have an effect on virus pathogenesis and immune response by changing the glycan shielding, or the possible immune decoy epitopes remains to be clarified. A distinct PRRSV population was reported that emerged by one single amino acid change in the GP5 ectodomain after infection in pigs [49]. Furthermore, it was reported for lactate dehydrogenase virus (LDV) in mice that a small number of mutations in ORF5 can alter the tropism and the interaction of virus with neutralizing antibodies favoring viral persistence [50]. Viral persistence may also be favored by a single amino acid change in the C-terminus region of a viral protein if this modification, creates a novel motif selecting the retainment of virions to the endoplasmatic reticulum (ER) [49]. Production of viral infectious clones of the 07V063 and 13V117 isolates and site-directed mutations will shed new light in this field.

In summary: (1) two new PRRSV strains designated 13V091 and 13V117 were isolated from pigs showing respiratory distress. Pathogenic and genetic studies showed that 13V091 is the most pathogenic type 1 subtype 1 strain isolated up till now showing fever, breathing problems, high virus titers in nasal secretions, an increased percentage of lung lesions in young animals and an expanded cell tropism to Sn^−^ cells in nasal mucosa and other lymphoid tissues. (2) Despite the fact that 13V117 and 07V063 have only eight amino acid differences in their genomes, they showed contrasting clinical and virological outcomes. 13V117 was able to induce fever, high viremia, and respiratory problems at the early stages of infection and it was able to replicate in the nasal mucosa but not in the lymph nodes, whereas 07V063 showed only fever, a 10-fold lower viremia, low virus titers in nasal excretions, but higher virus titers in lymphoid tissues.

These are important findings in order to evaluate the virulence and the pathogenesis caused by different type 1 subtype 1 PRRSV strains, to control the respiratory disease caused by PRRSV in young animals, and to develop novel vaccines for the newly emerged PRRSV isolates.

## Additional files

Additional file 1:
**List of clinical scoring.** All the parameters were scored for each individual animal and a sum of total scoring was taken into account. Additional file 2:
**Detailed clinical scoring and body temperature of all animals.** Daily registration of clinical scores (CS) and body temperature (BT) for each individual animal of PRRSV-infected and control groups is shown. Additional file 3:
**Partial amino acid alignment of the highly diverse region of nsp2 is presented.** The numbering is counted from the first amino acid in the poly protein 1a (pp1a) encoded from ORF1a. Grey squares represent deletions. Additional file 4:
**Phylogenetic analysis of ORF5.** The tree was constructed by the Neighbor Joining algorithm with Bootstrap 1000 replicates. Bootstrap values above 69% are shown in percent. 13V091 and 13V117 are highlighted in red. Additional file 5:
**Pairwise amino acid comparisons between different type 1 PRRSV strains.** Each open reading frame was translated into amino acid sequences and compared.
