# Supplementary material for: Different clinical, virological, serological and tissue tropism outcomes of two new and one old Belgian type 1 subtype 1 porcine reproductive and respiratory virus (PRRSV) isolates
Source: Vet Res. 2015 Mar 21;46(1):37. doi: 10.1186/s13567-015-0166-3 (PMC4367851; doi:10.1186/s13567-015-0166-3)
Supplement: Additional file 1: — List of clinical scoring. All the parameters were scored for each individual animal and a sum of total scoring was taken into account. [file 13567_2015_166_MOESM1_ESM.pdf]

**Parameter**	**Criteria**	**Score**
Breathing	Normal (<40/minute)	0
	Increased frequency (40-60/minute), snoring, barely visible chest movement	1
	Increased frequency (60-80 per minute), snoring, distinct chest and abdominal movement	2
	Increased frequency (>80 per minute), problems breathing, panting, breathing through open mouth	3
Sneezing	No sneezing	0
	Mild (<2 in 3 minutes observation)	1
	Severe (>2 in 3 minutes observation)	2
Coughing	No coughing	0
	Mild (<2 in 3 minutes observation)	1
	Severe (>2 in 3 minutes observation)	2
Nasal discharge	No nasal discharge	0
	Clear nasal discharge	1
	Discolored nasal discharge	2
Liveliness	No abnormalities	0
	Slightly reduced	1
	Tired, gets up only when forced	2
	Dormant, will not stand up	3
Discoloration of the ears	Normal	0
	Discolored skin of the ears: red, pale, gray or yellow	1
	Blue-purple discoloration of the ears	2
	Ear necrosis	3
Eyes	Normal	0
	Conjuctivitis	1
	Non-purulent secretion	2
	Purulent secretion	3
	Peri-ocular oedema	4
Digestive tract	Normal	0
	Diarrhea	1
	Diarrhea with blood and/or fibrin	2
