# Supplementary figures and images for: Different clinical, virological, serological and tissue tropism outcomes of two new and one old Belgian type 1 subtype 1 porcine reproductive and respiratory virus (PRRSV) isolates
Source: Vet Res. 2015 Mar 21;46(1):37. doi: 10.1186/s13567-015-0166-3 (PMC4367851; doi:10.1186/s13567-015-0166-3)

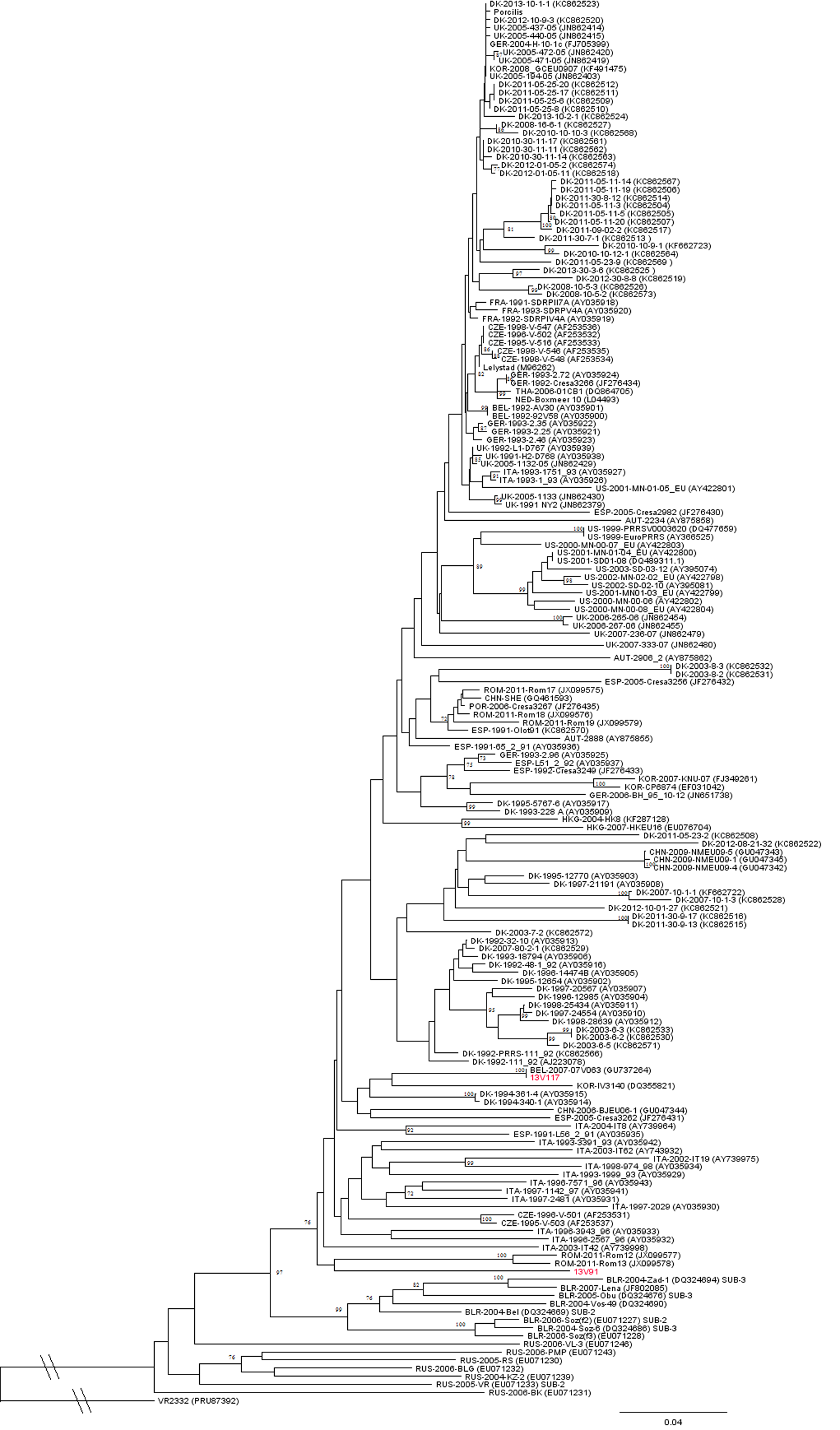

Supplement: Additional file 3: — Partial amino acid alignment of the highly diverse region of nsp2 is presented. The numbering is counted from the first amino acid in the poly protein 1a (pp1a) encoded from ORF1a. Grey squares represent deletions. [file 13567_2015_166_MOESM3_ESM.png]

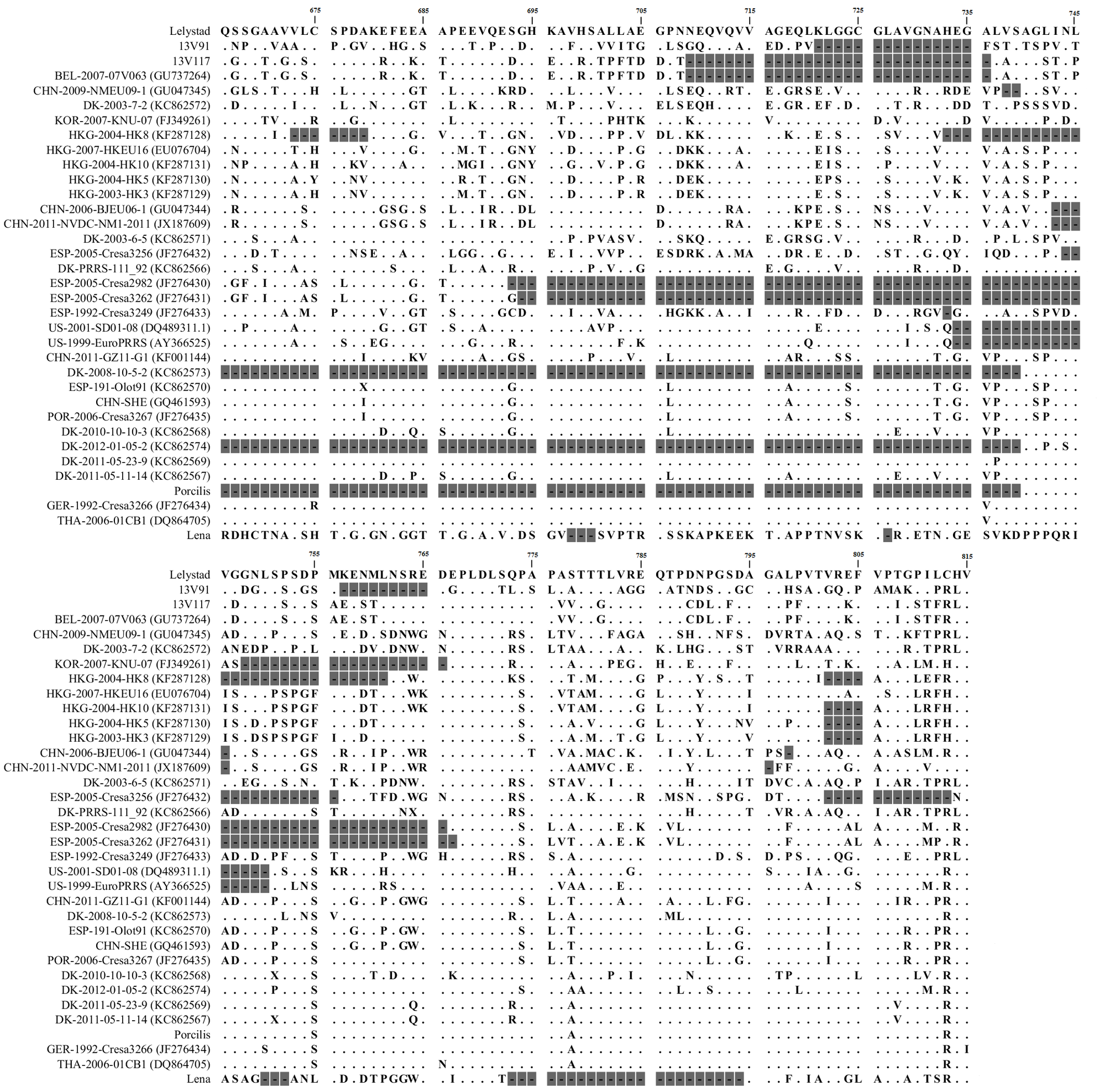

Supplement: Additional file 5: — Pairwise amino acid comparisons between different type 1 PRRSV strains. Each open reading frame was translated into amino acid sequences and compared. [file 13567_2015_166_MOESM5_ESM.pdf]
