# Supplementary material for: Different clinical, virological, serological and tissue tropism outcomes of two new and one old Belgian type 1 subtype 1 porcine reproductive and respiratory virus (PRRSV) isolates
Source: Vet Res. 2015 Mar 21;46(1):37. doi: 10.1186/s13567-015-0166-3 (PMC4367851; doi:10.1186/s13567-015-0166-3)
Supplement: Additional file 4: — Phylogenetic analysis of ORF5. The tree was constructed by the Neighbor Joining algorithm with Bootstrap 1000 replicates. Bootstrap values above 69% are shown in percent. 13V091 and 13V117 are highlighted in red. [file 13567_2015_166_MOESM4_ESM.png]

**13V091**	**Lelystad**	**07V063**	**13V117**	**Lena**
pp1a	86.77	85.47	85.71	77.94
nsp2	79.96	77.33	77.33	68.06
pp1b	94.81	93.74	93.74	91.63
GP2	89.16	88.76	88.76	88.76
E	95.71	95.71	95.71	94.29
GP3	82.64	79.25	78.87	74.34
GP4	85.25	83.61	83.06	78.80
GP5	88.06	88.06	88.06	82.09
M	89.6	93.06	93.06	90.75
N	86.72	84.38	84.38	84.38
**13V117**	**LV**	**07V063**	**13V091**	**Lena**
pp1a	88.81	99.83	85.71	78.64
nsp2	84.23	99.81	77.33	68.06
pp1b	95.54	99.87	93.74	91.43
GP2	93.57	100	88.76	92.77
E	95.71	100	95.71	95.71
GP3	88.68	99.62	78.87	76.23
GP4	86.89	99.45	83.06	80.06
GP5	91.04	100	88.06	81.59
M	93.06	100	93.06	92.49
N	90.62	100	84.38	83.59
